# Custom-made 3D printed subperiosteal titanium implants for the prosthetic restoration of the atrophic posterior mandible of elderly patients: a case series

**DOI:** 10.1186/s41205-019-0055-x

**Published:** 2020-01-08

**Authors:** Carlo Mangano, Andrea Bianchi, Francesco Guido Mangano, Jessica Dana, Marco Colombo, Ivan Solop, Oleg Admakin

**Affiliations:** 1 Department of Dental Sciences, University Vita Salute S. Raffaele, 20132 Milan, Italy; 2 Department of Periodontology and Implantology, Istituto Stomatologico Italiano, 20122 Milan, Italy; 3 Department of Prevention and Communal Dentistry, Sechenov First Moscow State Medical University, 119991 Moscow, Russia; 4 Private Practice, 6918 Lugano, Switzerland; 5 Private Practice, 21100 Varese, Italy

**Keywords:** Custom-made, Subperiosteal implants, Direct metal laser sintering (DMLS), Atrophic posterior mandible, Elderly patients

## Abstract

**Purpose:**

To present the application of custom-made 3D-printed subperiosteal implants for fixed prosthetic restoration of the atrophic posterior mandible of elderly patients.

**Methods:**

Between January 2017 and June 2018, all partially edentulous patients aged over 65 years, with two or more missing teeth in the posterior atrophic mandible, and who did not want to undergo bone regenerative procedures, were included in this study. These patients were rehabilitated with custom-made subperiosteal implants, designed from cone beam computed tomography (CBCT) and fabricated in titanium by means of direct metal laser sintering (DMLS). The outcome measures were fit and stability of the implants at placement, duration of the intervention, implant survival, and early and late complications. All patients were followed for 1 year after surgery.

**Results:**

Ten patients (four males, six females; mean age 69.6, SD ± 2.8, median 69, 95% CI 67.9–71.6) were included in the study. The fit of the implants was satisfactory, with a mean rating of 7 out of 10 (SD ± 1.6, median 7, 95% CI 6–8). Only two implants had insufficient fit, because of the presence of scattering in the CBCT; however, they were adapted to the sites during the interventions. The mean duration of the intervention was 44.3 min (SD ± 19.4, median 37, 95% CI 32.3–56.3). At the one-year follow-up, no implants were lost (survival rate 100%). One implant presented immediate postoperative complications with pain, discomfort and swelling, and two patients experienced late complications, having their provisional restorations fractured during the temporisation phase. All these complications were minor in nature, but the final complication rate amounted to 30% (three of ten patients).

**Conclusions:**

Although this study has limits (small patient sample and short follow-up), DMLS has proven to be an effective method for fabricating accurate subperiosteal implants, with high survival rates. This may represent an alternative treatment procedure in elderly patients with a severely atrophic posterior mandible, since it allows avoidance of regenerative bone therapies. Further studies are needed to confirm these outcomes.

## Background

Subperiosteal dental implants appeared in Sweden and the United States in the middle of the last century [1, 2]. Subperiosteal implants were custom-made fixtures, inserted below the periosteum, and stabilised by contact with the underlying bone, by means of fixation screws and the fibro-mucous tissue that covered them [2–4]. They were usually made of cobalt-chrome or titanium alloys and were prosthetised by means of transmucosal abutments that emerged inside the oral cavity [3, 5].

The technical fabrication of subperiosteal implants was complex, as it was necessary to capture a physical impression of the residual bone that was skeletonised, in a preliminary surgical session that caused significant patient discomfort [6, 7]. Then, during the surgical session to position them, these implants were far from precise, with the risk of unpredictable clinical results [7]; in fact, the need to adapt these implants during surgery could lead to long procedures, with increased risk of infections and complications [7, 8].

Subperiosteal implants were used for several years, but because of the difficulty in positioning them [6] and the high complication rates [7, 8], they were replaced by endosseous, root-form dental implants, introduced by Professor Brånemark from the University of Gothenburg [9].

Endosseous implants solved various issues associated with subperiosteal implants, and rapidly replaced them. More than 30 years of follow-up have shown that endosseous dental implants are a reliable and successful solution for the prosthetic restoration of partially [10] and totally edentulous patients [11], in the short [11] and long term [12].

A requirement for endosseous implant insertion is adequate bone quantity and quality. In the absence of adequate bone, three possible solutions presently exist. The first is to use reconstructive materials with techniques identified as onlay/inlay bone grafting [13], guided bone regeneration with non-resorbable [14] or resorbable membranes [15], alveolar ridge split [16], distraction osteogenesis [17] or sinus augmentation [18]. The issue with these techniques is the length of treatment, with the possibility of intra- and postoperative complications, due to the complexity of the procedures. In addition, they add economic costs for the patient [19]. The second option for inserting endosseous implants in unfavourable anatomical sites, without the aid of bone regeneration, is the use of short [20], narrow [21] or tilted implants [22]. Zygomatic [23] and pterygomaxillary implants [24] are also on the market, although less used in daily practice.

With the advent of digital technology, a new era in dentistry has begun [25]. From acquisition methods such as cone beam computed tomography (CBCT), which have considerably reduced the number of x-rays given to patients [26], to intraoral scanners [27], digital software, 3D printers and many other methods and materials [28], these technologies have simplified, improved and substantially sped up several procedures. Such technological advancement allows clinicians to see the world of dentistry in a completely different way, which is developing exponentially [25].

This digital revolution opens up new horizons, such as 3D printing and in particular direct metal laser sintering (DMLS) [29], which allows fabrication of custom-made meshes [30, 31] and even implants [32, 33] perfectly adapted to the patient’s specific anatomy.

This allows the opportunity to revisit some old concepts, such as the placement of subperiosteal implants, and reinterpret them in a new technological context based on consolidated anatomical and physiological principles [34–36]. The reduction of treatment to a single surgical session, lower costs for the patient and, above all, the precision that makes the method more predictable and safer in the short term have brought the attention of clinicians back to the use of subperiosteal implants, particularly for the management of complex atrophies such as in the posterior mandible of elderly patients [36, 37].

In severe posterior mandible bone resorption, when the patient does not want to undergo bone regeneration, modern digital technologies may represent a viable solution, with the possibility to fabricate custom-made subperiosteal implants perfectly adapted to their local morphology and anatomy [36, 37]. This is of particularly interest for elderly patients with special needs, who do not want or cannot undergo complex regenerative surgeries, but need a fixed prosthetic restoration [37].

The purpose of this case series was to show the clinical application of custom-made 3D-printed subperiosteal titanium implants for fixed prosthetic restoration of the atrophic posterior mandible of elderly patients.

## Methods

### Data acquisition

**Fig. 1:**
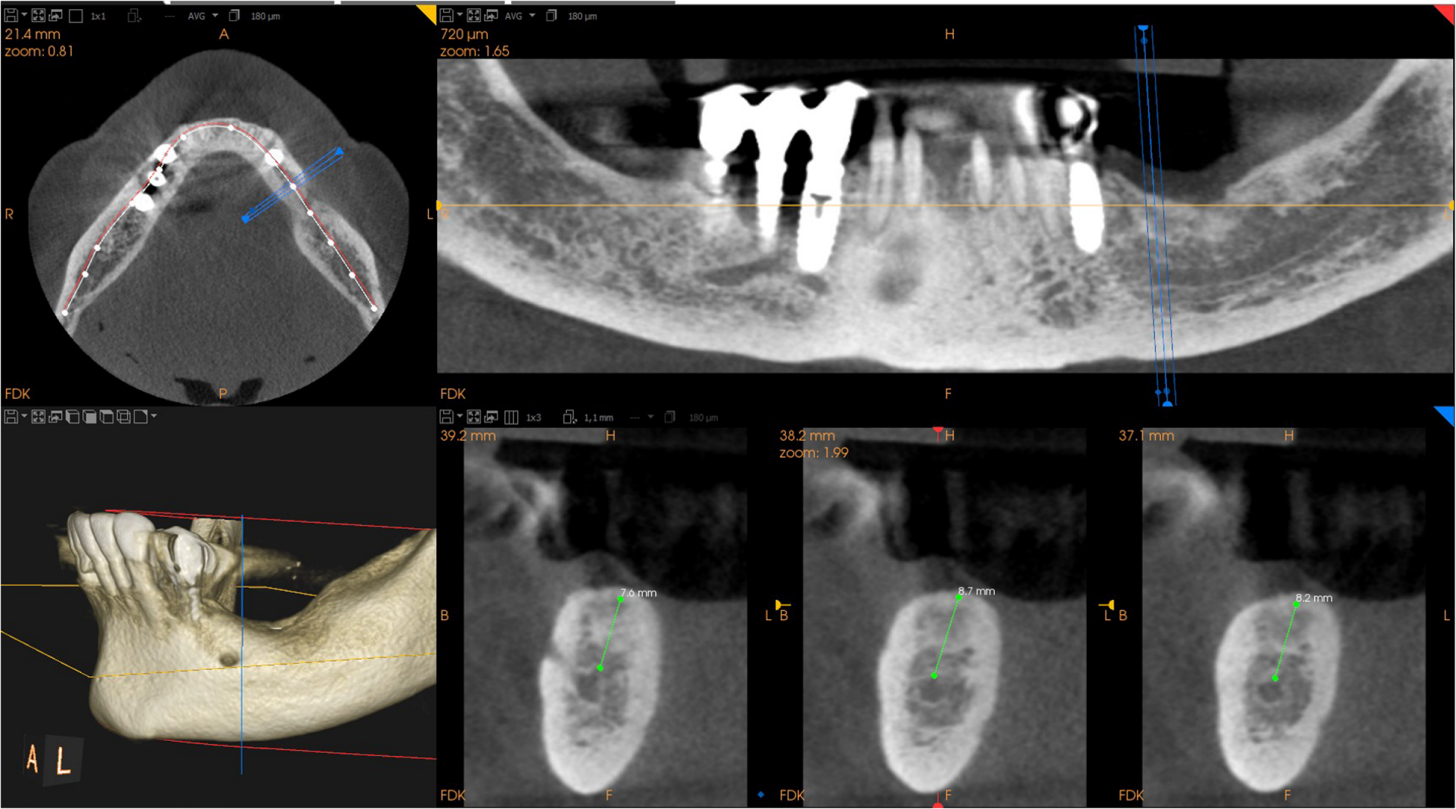
Initial CBCT (CS 9300®, Carestream Dental, Atlanta, GA, USA) that revealed a limited bone height (< 8 mm from the top of the crest to the inferior alveolar nerve) in the posterior left mandible. The 69 years old female patient was referred to our clinic and since she had experienced in the past failures with bone regenerative therapies, she did not want to undergo bone regeneration; the placement of a standard length endosseous implant was therefore not possible, and the only possibility was the placement of an extrashort (6 mm) endosseous implant

**Fig. 2:**
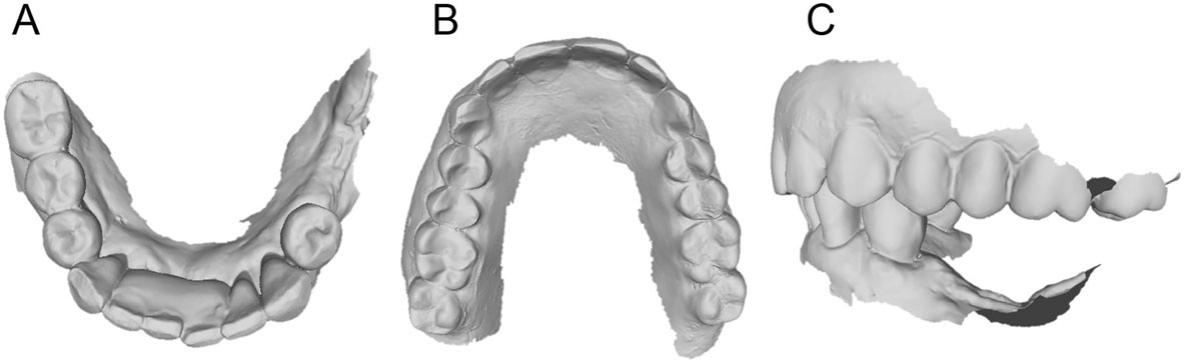
Intraoral scan (CS 3600®, Carestream Dental, Atlanta, GA, USA) of the patient’s arches. **a** Mandible, occlusal view; **b** maxilla, occlusal view; **c** occlusion, lateral view

Between January 2017 and June 2018, all partially edentulous patients with missing teeth in the posterior mandible, who were considered for possible inclusion in this study, received a radiographic evaluation. The radiographic evaluation took place with an orthopantomography (OPT), and ended with a CBCT (CS 9300®, Carestream Dental, Atlanta, GA, USA) for correct 3D evaluation of the height, thickness and angulation of the residual bone (Fig. 1). Specific fields of view were selected (5 × 5 cm or 10 × 5 cm, with a slice thickness of 90 to 200 μm, respectively) to reduce patients’ exposure to radiation and to gather high-resolution digital imaging and communication in medicine (DICOM) data. However, since the final purpose was prosthetic rehabilitation, and to properly understand patients’ needs and occlusal requirements, patients’ arches were also scanned using a powerful intraoral scanner (CS 3600®, Carestream Dental, Atlanta, GA, USA) (Fig. 2). Data were saved as stereolithographic (.STL) files, and used to prepare a diagnostic wax-up with computer-assisted design (CAD) software (DentalCad®, Darmstad, Exocad, Germany). This diagnostic wax-up allowed building the shape and size of the teeth that would be part of the future cemented prosthesis, and understanding the ideal position of the future prosthetic abutments.

### Inclusion and exclusion criteria

The inclusion criteria for enrolment in the study were:

- age over 65 years
- good systemic and oral health
- acceptable oral hygiene
- partially edentulous mandible, with two or more teeth missing in the posterior sectors and marked atrophy that disallowed insertion of standard size implants (length ≥ 10 mm)
- willingness not to undergo regenerative bone surgery
- willingness to attend the follow-up control visits.

The exclusion criteria for this study were:

- age under 65 years
- systemic pathologies or pharmacological therapies that could contraindicate the intervention (such as immunocompromised states, non-compensated diabetes, tumours of the head and neck, or treatment with oral or parenteral bisphosphonates)
- inadequate oral hygiene
- smoking habit
- bruxism
- completely edentulous mandible, or partially edentulous mandible in the posterior sectors with bony bases allowing insertion of implants of standard dimensions (length ≥ 10 mm) without the risk of damaging nervous structures
- lack of willingness to undergo the necessary control visits.

The study was approved by the local Ethics Committee at Sechenov University (Moscow) with number #8819 and carried out in full compliance with the 1975 Declaration of Helsinki on patient rights (2008 revision).

### Implant design

**Fig. 3:**
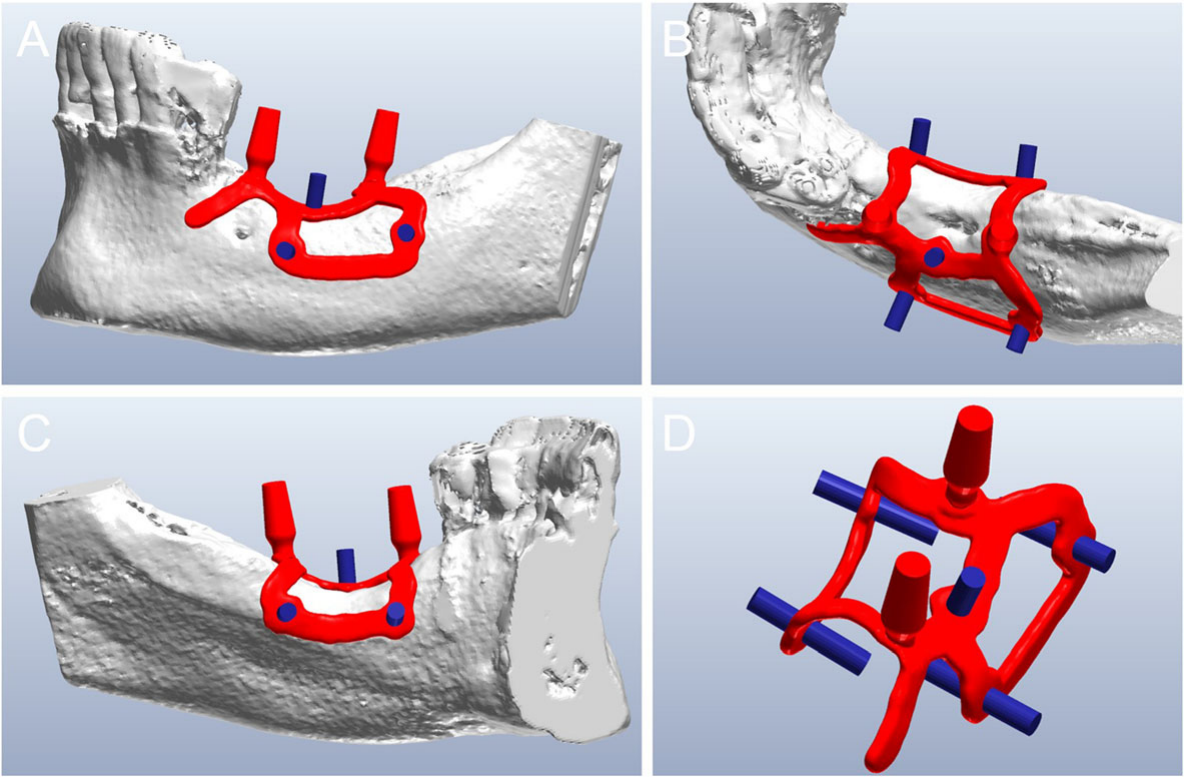
The custom-made subperiosteal implant is designed in a CAD software (Meshmixer®, Autodesk, San Rafael, CA, USA). **a** The implant with the integral abutments and the fixation screws, buccal view; **b** occlusal view; **c** lingual view; **d** detail of the axes for the fixation screws

**Fig. 4:**
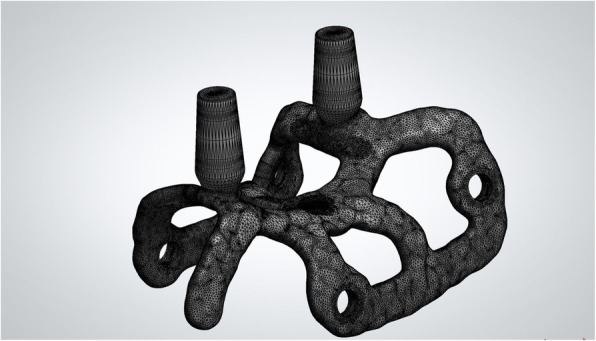
Detail of the mesh of the implant, ready for fabrication via DMLS

The DICOM data obtained from the CBCT were extracted and imported into software where the residual anatomy of the patient’s bone was reconstructed in 3D (Mimics®, Materialise, Leuven, Belgium), and the file saved as. STL. In this phase, care was taken to investigate the position of noble structures (such as the inferior alveolar nerve), and to select the proper threshold values, to best define the cortical walls of the residual bone. The best position for the fixation screw was also evaluated. The. STL file of the 3D bone reconstruction was then imported to reverse engineering software (Studio 2012®, Geomagics, Morrisville, NC, USA) where a cleaning operation was carried out, with elimination of scattering where present and sharp edges or mesh errors; furthermore, the. STL density was reduced. This 3D reconstruction was then aligned with the. STL files obtained from the intraoral scan of the patient’s arch, and with the diagnostic wax-up, with the ultimate aim of having a file with the whole information of the patient. This allowed better understanding of the ideal prosthetic emergence profile, and therefore how to properly design the implant. All these files were imported to another CAD software (Meshmixer®, Autodesk, San Rafael, CA, USA), where the prosthetic abutments and osteosynthesis screws for implant stabilisation were designed. The subperiosteal implant framework was drawn, connecting all the structures through a series of Boolean operations (Fig. 3). The. STL of the final modelling was then exported again to Studio 2012® (Geomagics, Morrisville, NC, USA) for a final edge correction, final quality control and file regularisation (Fig. 4). The implant file was then ready for fabrication.

### Implant fabrication

**Fig. 5:**
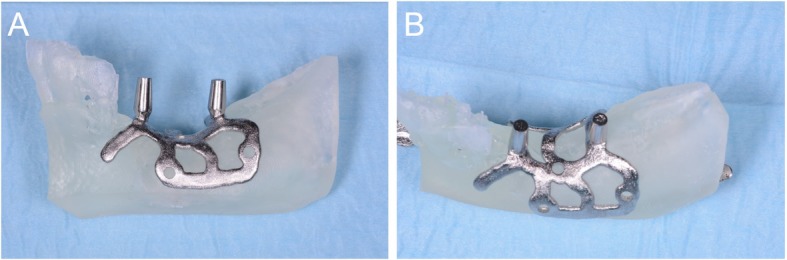
A replica of the implant (Iuxta3D®, BTK, Dueville, Vicenza, Italy), fabricated via DMLS, is tested in a 3D printed copy of the patient’s mandible, printed with a stereolithographic (SLA) 3D printer (3500PD®, DWS Systems, Thiene, Vicenza, Italy). The adaptation looks perfect. **a** Buccal view; **b** occlusal view

A DMLS machine was used (ProX-DMP100®, 3D System, Rock Hill, SC, USA) to print the subperiosteal implants. This machine was able to build the custom-made subperiosteal implant exactly as designed, starting from titanium grade 5 micro-powders, layer by layer, using a powerful laser beam (50 W fibre laser with a wavelength of 1070 nm), with layer size of 20 μm. The build envelope capacity of the machine was 100 × 100 × 80 mm. The porous and chemically pure implant was washed with organic acids, decontaminated and sterilized. At the same time, the 3D bone reconstruction and a replica of the subperiosteal implant were printed in resin using a 3D printer (ProJet 3510 MP®, 3D system, Rock Hill, SC, USA). The 3D model of the bone, printed with a stereolithographic (SLA) printer (3500PD®, DWS systems, Thiene, Vicenza, Italy), was used to verify the anatomy, and to control the fit of a replica of the DMLS subperiosteal implant (Fig. 5); the implant replica was printed in biocompatible resin, to help the surgeon in preparation of the flap and access for fixation of the implants intraoperatively.

### Surgery

**Fig. 6:**
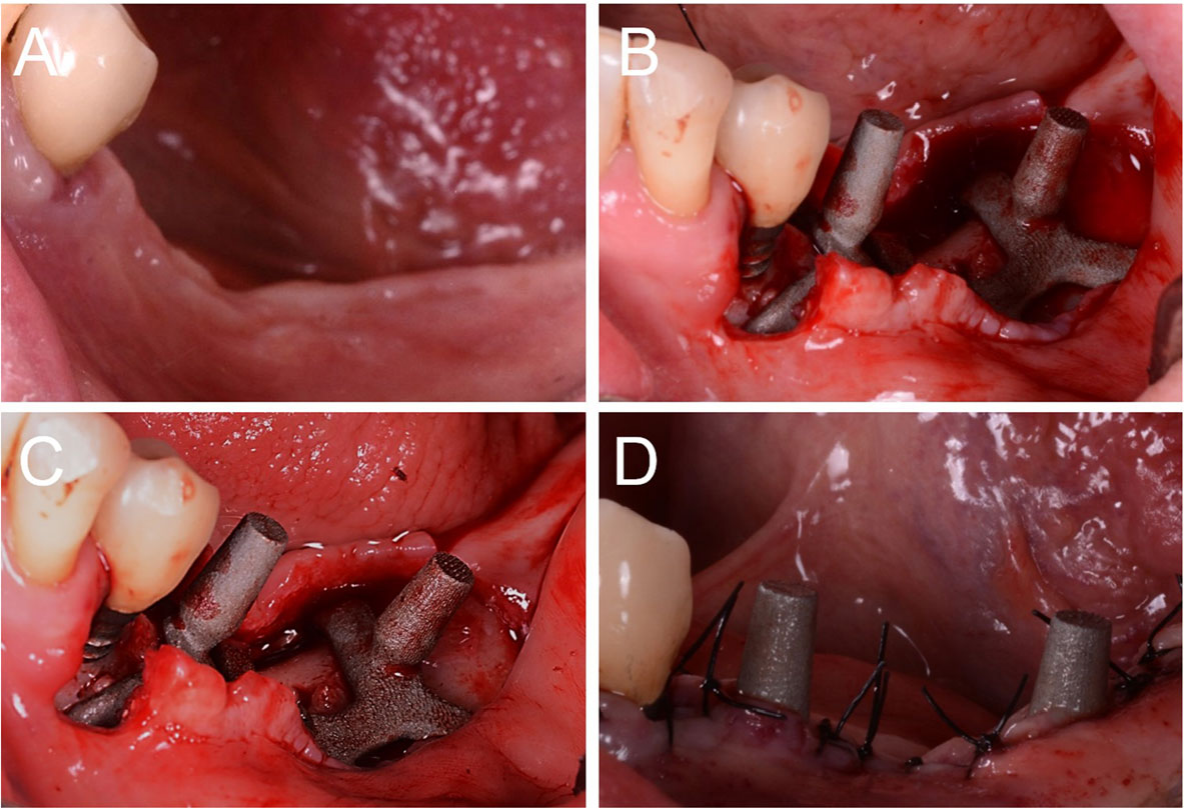
Surgery on patient. **a** Pre-surgical picture; **b** the mucoperiosteal flap is raised and the implant (Iuxta3D®, BTK, Dueville, Vicenza, Italy) is manually adapted on the anatomical site; (**c**) the implant is fixed on site by means of the fixation screws; **d** sutures around the abutments

**Fig. 7:**
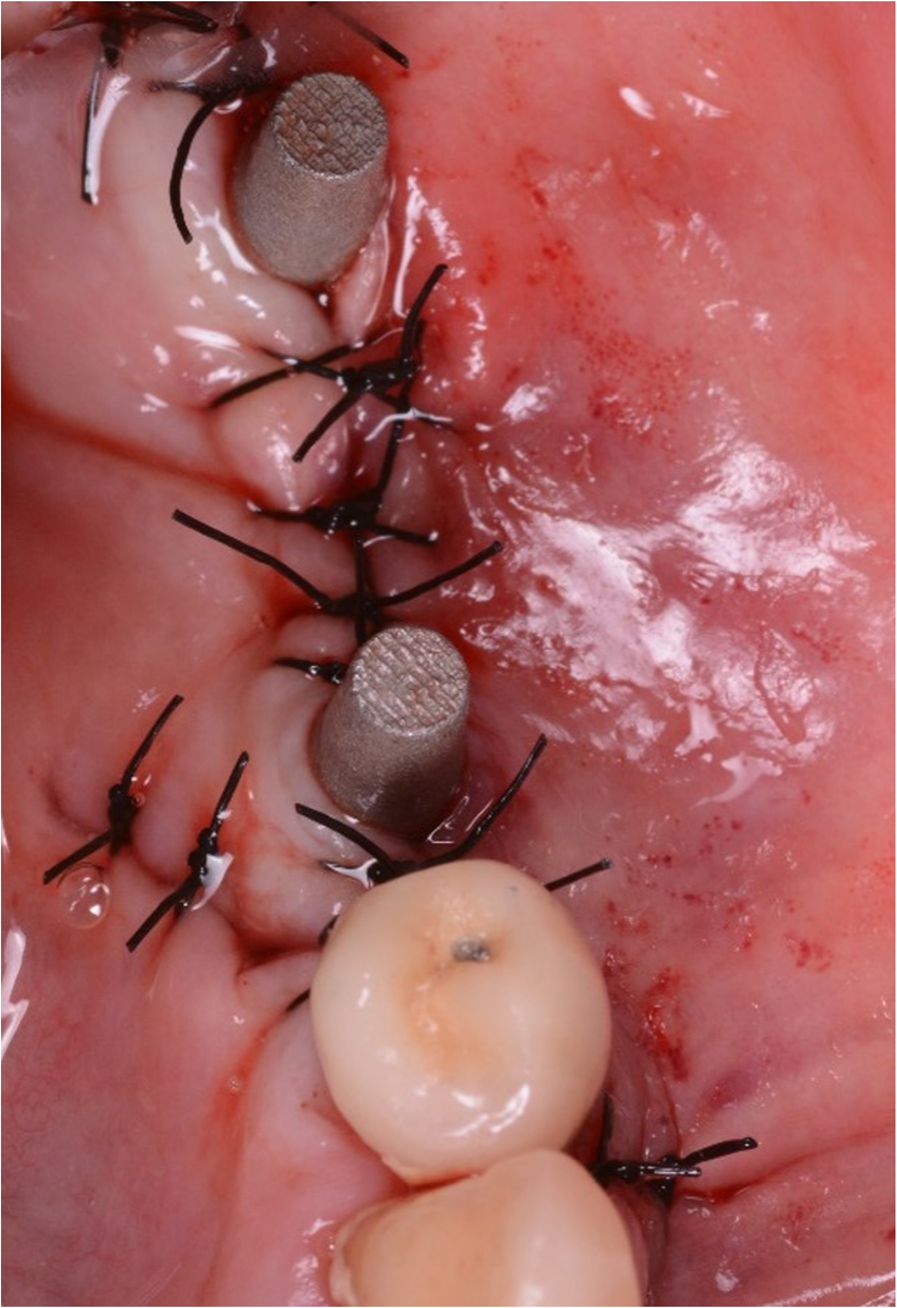
Sutures, occlusal view

Once all materials were ready, the surgical phase could start, using local anaesthesia (4% articaine, 1:100,000 adrenaline). A crestal incision was performed, delimited by mesial and distal release incisions, and a full-thickness flap was raised, for a complete view of the implant site. The implant replica was used to prepare the flap, to verify the perfect adaptation of the implant intraoperatively, and for preparation (drilling) of access for the miniscrews to be used for fixation of the grid (Fig. 6). Once the site was prepared, the DMLS implant (Iuxta3D®, BTK, Dueville, Vicenza, Italy) was removed from its sterile pack, placed on the site to verify its adaptation on the residual bone, and then fixed with the aid of osteosynthetic miniscrews. The surgical site was sutured, and using periosteum release incisions, flap passivation was performed to cover the whole section and obtain first-intention healing. In suturing, particular attention was paid to avoid excessive tension, maintaining a correct amount of keratinised gingiva around the emerging abutments, which is necessary for clinical success, both surgically and prosthetically (Fig. 7). At the end of surgery, antibiotic therapy was prescribed (amoxicillin plus clavulanic acid 1 g every 12 h for 6 days), along with painkillers (ibuprofen 600 mg to be taken for the following two to 3 days) and antibacterial therapy (mouthrinses of 0.12% chlorhexidine, two to three times per day, for five to 6 days).

### Prosthesis

**Fig. 8:**
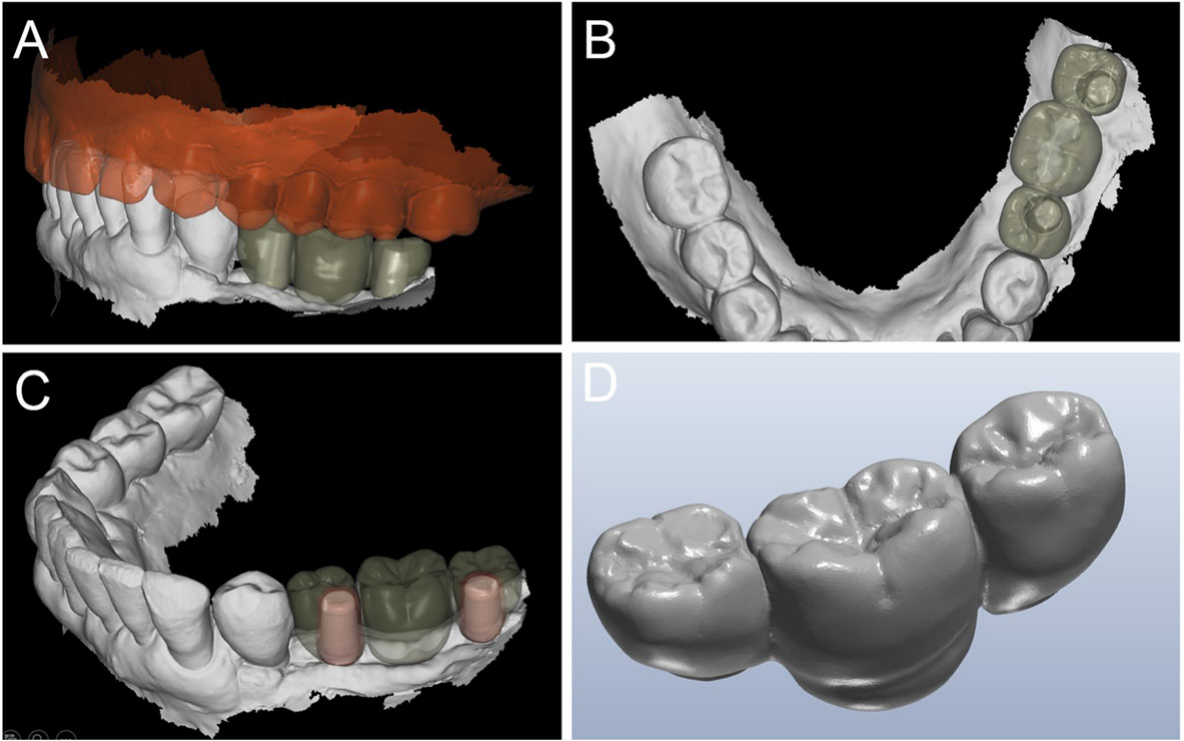
CAD design (DentalCad®, Darmstad, Exocad, Germany) of the pre-milled restoration. **a** Buccal view; **b** occlusal view; **c** perspective view; **d** the pre-milled restoration

**Fig. 9:**
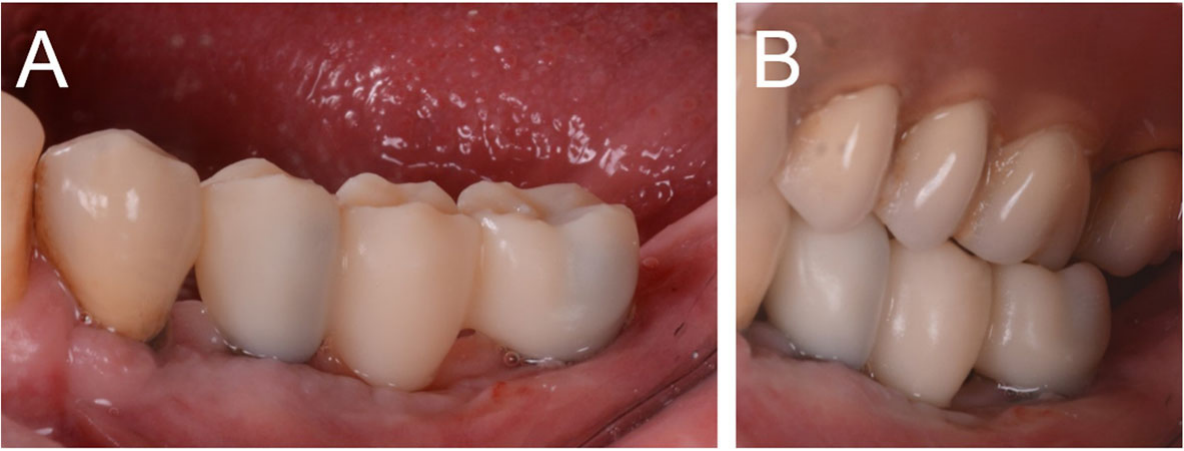
The pre-milled provisional restoration. **a** Buccal view; **b** the pre-milled restoration relined and adapted in occlusion

**Fig. 10:**
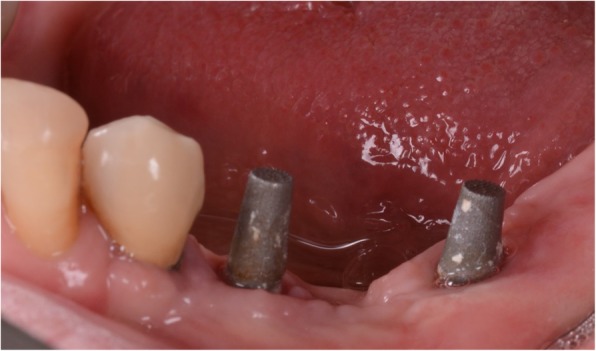
Control after 2 months, at the end of the provisionalization

**Fig. 11:**
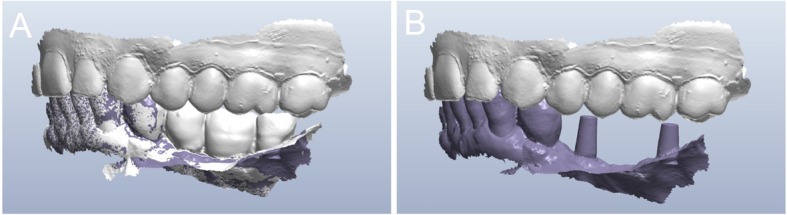
Second intraoral scan, taken at the end of the provisionalization. **a** Scan with the provisionals in situ, useful as a reference for occlusion; **b** direct intraoral scan of the emerging abutments

**Fig. 12:**
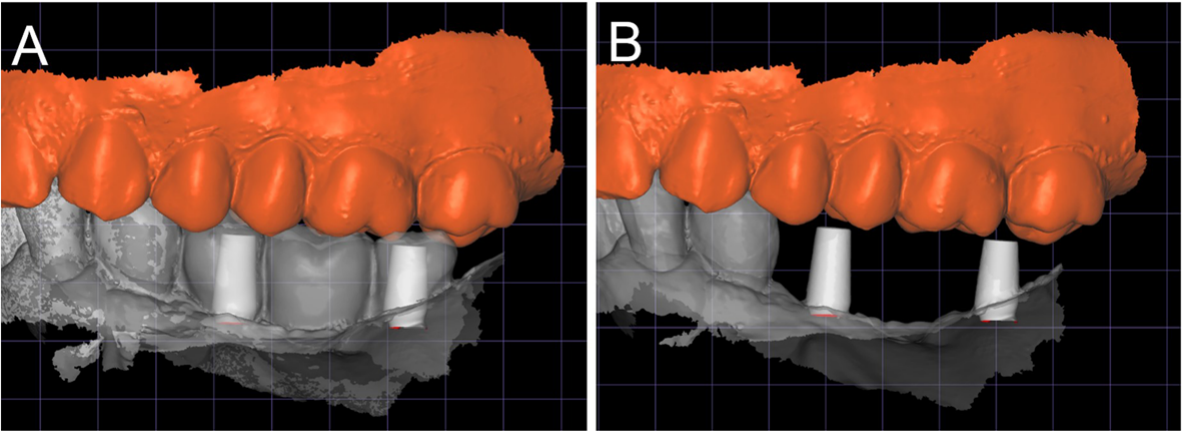
The final scan was sent to the dental technician, who once again replaced the meshes of the abutments with the original CAD files of the implant in the same aforementioned CAD software (DentalCad®, Darmstad, Exocad, Germany). **a** The meshes of the emerging abutments are replaced by the original CAD design; **b** the original CAD files of the abutments in position, after alignment

**Fig. 13:**
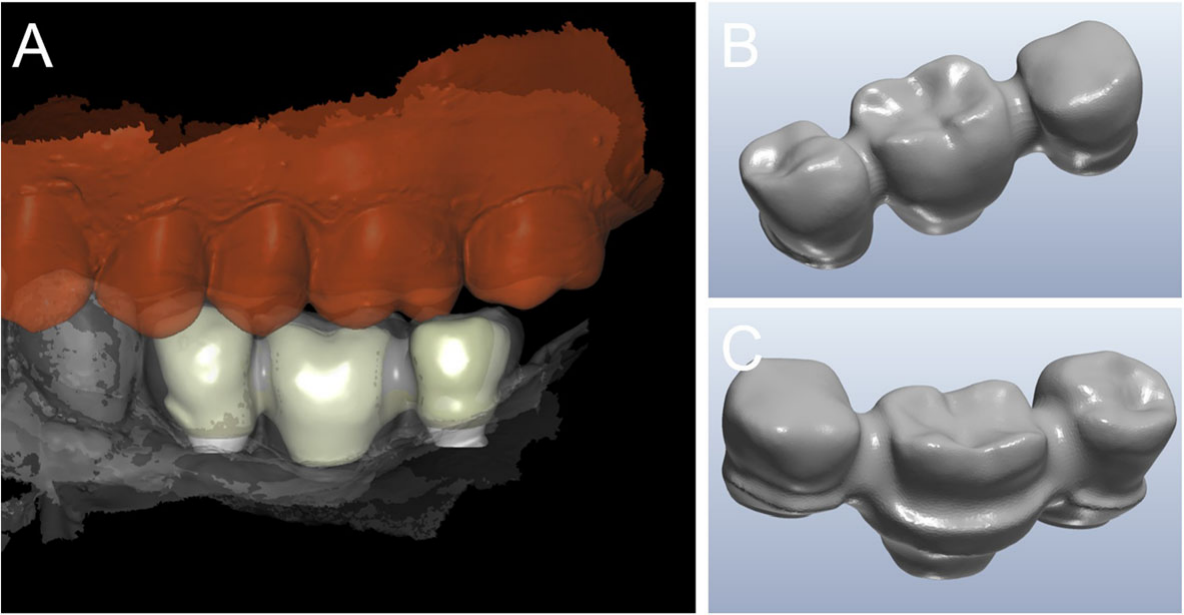
Final CAD project of the zirconia framework. **a** Buccal view of the framework; **b** the zirconia structure, buccal view; **c** the zirconia structure, lingual view

**Fig. 14:**
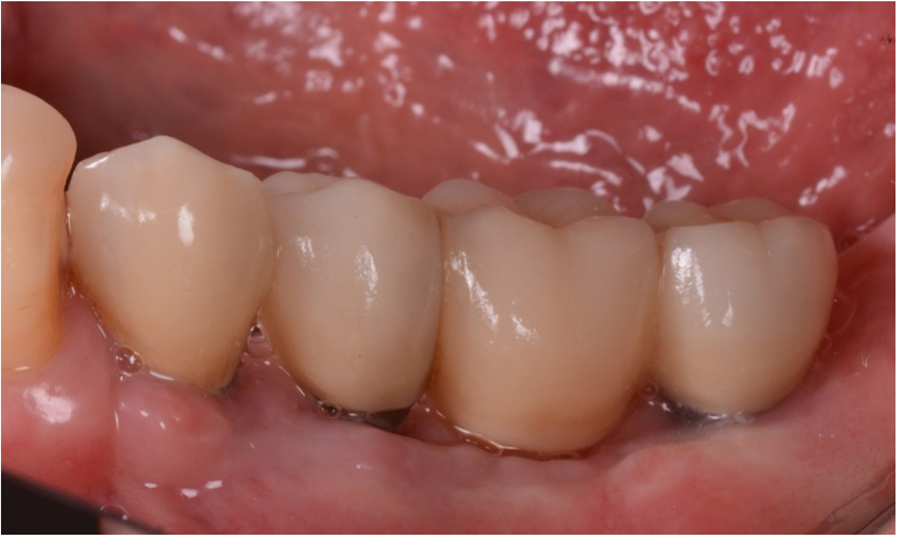
The final zirconia-ceramic bridge at delivery

Ten days after surgery, at suture removal, the prosthetic phase started with the delivery of the pre-milled computer-assisted design and manufacturing (CAD/CAM) temporary restoration in resin (Fig. 8). This restoration, milled in polymethyl-methacrylate (PMMA) with a smart desktop milling machine (DWX-4®, Roland, Ascoli Piceno, Italy), was cemented after careful adaptation in occlusion; care was taken to obtain excellent interproximal contact points (Fig. 9). A couple of weeks later, after soft tissue healing and sutures removal, a second intraoral impression was taken using an intraoral scanner (CS 3600®, Carestream Dental, Atlanta, GA, USA) for the preparation of a second provisional with higher precision. The. STL data from this scan were sent to the dental laboratory, where the meshes of the emerging abutments were replaced with the original CAD files of the same elements, taken from the original implant design. This allowed the dental technician to model the high-precision second provisionals. These second provisionals, again milled in PMMA with a desktop machine (DWX-4®, Roland, Ascoli Piceno, Italy), were characterized and cemented. They remained in situ for a period of 2 months, then were replaced by the final restorations in zirconia-ceramic. Another intraoral scan (CS 3600®, Carestream Dental, Atlanta, GA, USA) was taken of the emerging abutments in situ, before and after removal of the provisionals (Figs. 10, 11). This final scan was sent to the dental technician, who once again replaced the meshes of the abutments with the original CAD files of the implant in the same CAD software (DentalCad®, Darmstad, Exocad, Germany) (Fig. 12), modelling a zirconia framework (Fig. 13). The definitive zirconia framework was produced by milling with a powerful five-axis milling machine (Roland DWX-50®, Roland Easy Shape, Ascoli Piceno, Italy), subsequently sintered in an oven (Tabeo®, Mihm-Vogt, Stutensee, Germany), characterized and ready for ceramic stratification. The technician generated 3D models of the mandible and maxilla and printed them using a powerful 3D printer (3500PD®, DWS, Thiene, Vicenza, Italy), and was able to add ceramic on the zirconia framework. The final zirconia-ceramic bridge was then delivered to the patient (Fig. 14), carefully controlled in occlusion and for the quality of interproximal contact points and colour. Cementation was carried out with a temporary cement (Tempbond®, Kerr, Orange, CA, USA). The patient was enrolled in an annual recall program, based on two- to three-yearly appointment for professional oral hygiene.

### Outcome measures

The outcome measures from this study were fit and stability of the implants, duration of the intervention, implant survival, and early and late complications.

#### Fit of the implants

A rating from 0 to 10 was given by the surgeon during the intervention, to rate the fit of the DMLS subperiosteal implants to the corresponding bone anatomy. Values ​​from 0 to 5 indicated a bad or insufficient adaptation, 6 a barely sufficient adaptation, and 7 to 10 a good to excellent adaptation and fit.

#### Duration of the intervention

The duration of the intervention was monitored by the chair assistant, from local anaesthesia to sutures. It was measured in minutes and reported in the patient’s record.

#### Implant survival

All subperiosteal implants that were correctly functioning at the one-year follow-up session were considered successful. Implants that were lost were considered failed. Causes for implant failure could be incorrect adaptation during surgery and consequent mobilisation or instability of the implant, implant fracture, infection, or loss of bone support in the absence of infection.

#### Early complications

Any immediate postoperative complications or secondary issues such as pain, swelling, oedema or bleeding arising within 2 weeks after the surgery, and before the placement of the first provisional restoration, were classified as early complications. These complications were biological in nature.

#### Late complications

Any biological or prosthetic complications that occurred between delivery of the first prosthetic restoration and the one-year follow-up were classified as late complications. These complications could be biological or prosthetic in nature. Late biological complications could include severe and/or recurrent infections, with exudation or suppuration, pain, swelling, or pus formation, with or without radiographic evidence of bone loss. Late prosthetic complications could include technical complications that afflicted the temporary restorations (e.g. fractures of the acrylic resin) or the final definitive ones (e.g. fractures or chipping of the zirconia-ceramic restorations).

### Statistical analysis

Data were collected by an independent examiner. Descriptive statistics were performed for patient demographics (gender and age at surgery). Absolute and relative frequency distributions were calculated for qualitative variables (fit and stability of implants, implant survival and complications), while means, standard deviations (SD), medians and confidence intervals (CI 95%) were found for quantitative variables (age at surgery, fit of implants and duration of intervention). All variables were calculated at the patient level.

## Results

Between January 2017 and June 2018, 15 partially edentulous patients with missing teeth in the posterior mandible were considered for inclusion in this study, for treatment with subperiosteal DMLS implants. Five were excluded: three were under 65 years old and two were smokers. Therefore, ten patients (four males and six females) were included. These patients were aged between 68 and 75 years (mean age 69.6, SD ± 2.8, median 69, 95% CI 67.9–71.6).

With regard to the study outcomes, the fit of the implants was extremely satisfactory, with a mean rating of 7 out of 10 (SD ± 1.6, median 7, 95% CI 6–8). Only two implants had an insufficient fit (with values of 4 and 5), mainly because of the presence of scattering from neighbouring crowns or teeth that interfered with the correct thresholding process. However, these implants were adapted to the surgical sites during the interventions.

The mean duration of the intervention was 44.3 min (SD ± 19.4; median 37; 95% CI 32.3–56.3). However, this result was deeply influenced by the two cases in which the adaptation was not fully satisfactory, which required respectively 85 and 67 min from anaesthesia to sutures.

**Fig. 15:**
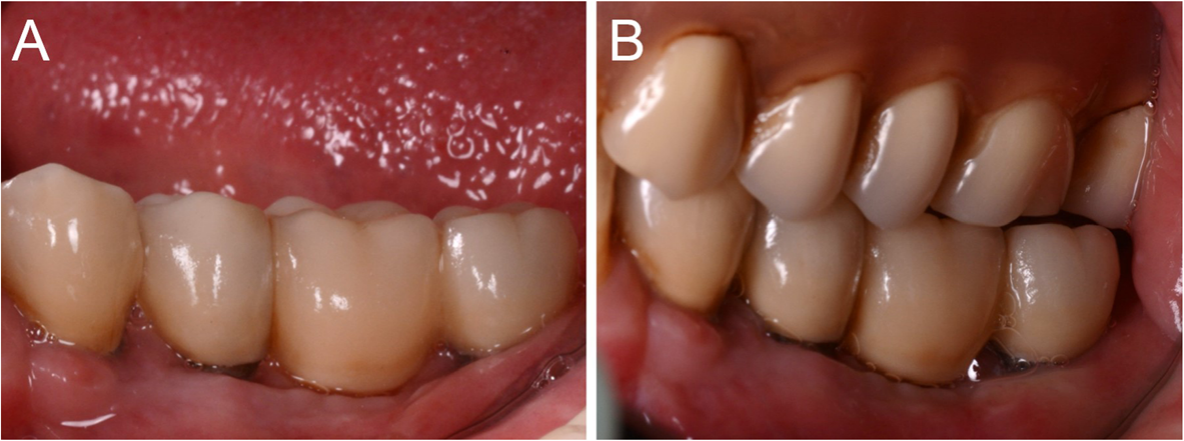
The zirconia-ceramic restoration at the 1-year follow-up control. **a** Buccal view; **b** View of the restoration in occlusion

**Fig. 16:**
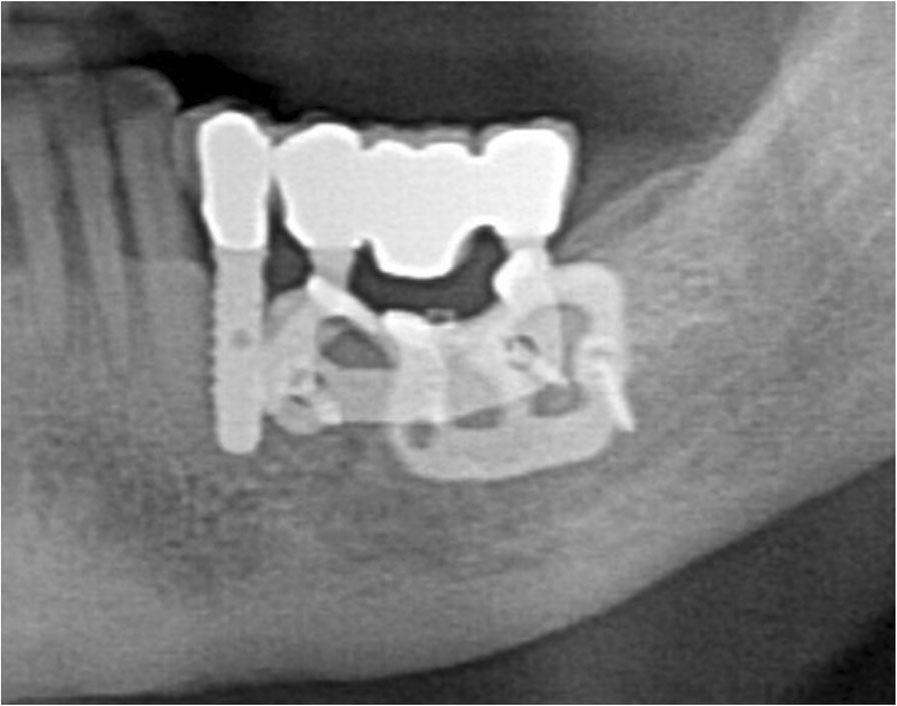
The zirconia-ceramic restoration at the 1-year follow-up control, radiographic control

At the one-year follow-up, no implants were lost, for a survival rate of 100% (Figs. 15, 16). However, one implant presented immediate postoperative complications, with pain and discomfort associated with swelling after the placement, and two patients experienced late complications. Among these, two patients had their provisional restorations fractured during the temporisation phase. However, when the provisionals were replaced by the final zirconia-ceramic restorations, no further prosthetic complications were reported. The incidence of early complications amounted to 10% (one of 10 patients), while the incidence of late complications was 20% (two of 10 patients). Although all these complications were minor in nature, an overall incidence of 30% complications was reported for these patients at the one-year follow-up control.

## Discussion

Subperiosteal implants have existed for many years and, until the advent of modern implantology *ad modum* Brånemark, were a solution for the prosthetic rehabilitation of partially and completely edentulous patients [1–5, 38–40]. Although some scientific works demonstrated long-term survival of these implants [41–43], and that osseointegration was indeed possible [42, 43], for various reasons they were abandoned and replaced in modern implantology with endosseous implants [6–8, 42, 43].

The endosseous implants proposed by Brånemark overcame several problems of subperiosteal implants, such as the need for two surgical sessions, with the necessity to skeletonise the patient and take a physical impression of the edentulous part, the productive limits of which caused difficulties in positioning and stabilisation, as well as the high incidence of postoperative failures and problems, and considerable patient discomfort [8–12].

To position endosseous implants, adequate bone volume is necessary [19]; unfortunately, bone volume is not always adequate, particularly in the posterior mandible of elderly patients. Moreover, this site is one of the most difficult to regenerate with conventional techniques [19, 44]. Bone regenerative techniques, although successful in many cases, entail an infection risk, may involve complications, and certainly increase cost and duration of therapy [44, 45]. In some elderly patients with severe bone atrophy, performing bone regeneration can be risky. For these patients, treatment can be difficult due to compromised general health, overly invasive procedures and higher costs. The use of short [20] and narrow [21] implants may therefore represent an alternative option, but if the bone volume has undergone significant contraction in both height and thickness, placing endosseous implants can be impossible.

For all these patients, and in all these situations, subperiosteal implants may be an alternative, due to the digital revolution and the advent of modern digital technologies in dentistry [34–37, 46]. CBCT allows acquisition of 3D data of the patient’s residual bone volume, with considerable accuracy, and a low dose of radiation directed exclusively at the area of ​​interest [27, 47]. The data acquired with the CBCT is then re-elaborated with appropriate reconstruction software, which allows generation of a virtual bone model of the area of ​​interest [34–37]. Capturing an optical impression of the dentate arches allows modelling of a virtual diagnostic wax-up. Finally, the superimposition of bone and dental models, together with the virtual wax-up, makes it possible to design and model custom-made subperiosteal implants, designed and conceived specifically for the patient’s needs, both as a bone-supported structure and as a prosthetic emergency [34–37]. These 3D-printed implants represent a possible alternative solution for the rehabilitation of the atrophic posterior mandible of elderly patients who do not want or cannot undergo traditional regenerative techniques, preparatory to insertion of classical endosseous implants [34–37].

In particular, increasingly advanced DMLS techniques allow revisiting the old concept of subperiosteal implants. DMLS is a technique arose in the late 1990s in Germany, which has since been gaining importance in dentistry [29]. It is an additive manufacturing (AM) technique that uses a high-power laser to melt metallic powders together [29]. The procedure involves construction of a stratified 3D model, layer by layer. The CAD file is sliced into thin layers, creating a two-dimensional images series that, laced together, create the three-dimensionality of the product [29]. The powder of each layer is then selectively melted by the laser beam, and the process is continuously repeated layer by layer, until completion of the device. With DMLS, there is almost no limitation in the fabrication of complex objects, such as porous, hollow objects with interconnections, tunnels and crevices. It is possible to manufacture titanium or titanium alloy implants, whether standard endosseous [29] or custom-made subperiosteal [34–37].

Surovas [35] demonstrated the feasibility and economic sustainability of the design and manufacture via DMLS of a custom-made subperiosteal implant in titanium alloy, able to adapt perfectly to the 3D-printed bone model. The steps for the design and fabrication of the custom-made implants were computerized tomography (CT) scan, CT data processing, 3D virtual model creation, modelling technique for custom implant, and data file preparation for 3D printing [35]. The custom-made subperiosteal implant was then fabricated in Ti6Al4V (a type 5 titanium alloy) using DMLS [35].

Cohen et al. [36] developed custom-made subperiosteal Ti6Al4V devices produced by AM and post-fabrication osteogenic micro- and nano-scale surface texture modification. The porous surface of these implants had the potential to stimulate human osteoblasts to produce osteogenic factors, and a high bone-to-implant contact was found for DMLS disks implanted in the rat calvaria and in the rabbit tibia [36]. When implanted in the human posterior mandible, three- and eight-month postoperative images showed new bone formation and osseointegration. The implants were stable and successful under function. These data are not surprising, given that the highly porous surface of titanium laser sintering implants has amply demonstrated in histological and histomorphometric human studies high levels of bone contact and osseointegration, with bone incorporation into the pores, in the posterior maxilla 2 months after insertion [48–50]. The possibility of obtaining a porous surface capable of stimulating bone formation represents one of the possible advantages of manufacturing subperiosteal implants with DMLS, and a major difference from traditional subperiosteal implants, which were fused and therefore presented a smooth surface.

In a retrospective clinical study, Cerea et al. [34] presented an analogue–digital technique for fabricating custom-made subperiosteal implants, and reported on the survival and complication rates encountered when using these fixtures. In total, 70 partially or completely edentulous patients were included in the study and treated with custom-made DMLS subperiosteal implants, in both the maxilla and the mandible [34]. These implants were designed in an analogue way by the surgeon, directly on 3D models, then digitally designed and fabricated via DMLS. At two-year follow-up, three implants were lost due to recurrent, untreatable infections; the implant survival rate was 95.8% [34]. With regard to complications, four patients had pain, discomfort or swelling after implant placement, giving an incidence of immediate postoperative complications of 5.7% [34]. During the follow-up period, one patient experienced recurrent infections, representing an incidence of biological complications of 1.4% [34]. The rate of prosthetic complications amounted to 8.9% [34]. The authors concluded that application of custom-made DMLS titanium subperiosteal implants can represent a successful strategy for the prosthetic restoration of patients with severe bone deficiencies, and an alternative to conventional bone regenerative techniques.

The clinical study of Cerea et al. [34] has the highest number of enrolled patients and the longest follow-up, though it should be noted that the process for fabrication of the implants was hybrid (analogue–digital) and all implants underwent an electropolishing treatment, capable of transforming the porous surface of the laser-sintered implants into a smooth one. This can radically change the response of hard and soft tissues to the implant. Moreover, the study included different categories of patients (including completely edentulous), with different fixation screws and in the maxillary area.

In our present study we have focused our attention only on partially edentulous patients, and in particular on the rehabilitation of the posterior mandible. Our case series showed positive clinical outcomes with the use of subperiosteal custom-made DMLS implants. First, the overall accuracy of the implants was excellent. Only two implants had an insufficient fit (with values of 4 and 5 rated by the surgeon), because of the presence of scattering from neighbouring crowns or teeth in the original CBCT that interfered with the thresholding process. However, these implants were adapted to the surgical sites during the interventions.

In our present study, the whole fabrication processes (from the intraoral scans and CBCT to the surgery) took approximately 2–3 weeks. The mean duration of the surgery was 44.3 min. Notably, this result was influenced by the two aforementioned cases in which adaptation was not fully satisfactory (those cases required respectively 85 and 67 min, from anaesthesia to sutures).

Finally, in our study, all implants survived at 1 year after placement. Immediate postoperative complications had a low incidence (10%), were minor in nature, and resolved in a few days with pain-relieving and antibiotic therapies. The low incidence of complications was a direct result of the perfect fit of the custom-made implants to the patient’s residual anatomy; the surgical procedures were simplified and sped up, inducing a more comfortable result.

The main advantages of our fully digital technique, compared to the conventional analogue technique used in the past for fabrication of subperiosteal implants, relies on the accuracy of the implants. This increases considerably the correspondence between the implant structure and the underlying bone, eliminating the need for a surgical session to capture a physical impression of the bone, reducing non-fitting problems and distributing the load more evenly. The excellent accuracy speeds up and simplifies the surgery, reducing the risk of bacterial contamination or infection. In addition, compared to the previously published study of Cerea et al. [34], in our present study the design and fabrication of the implants was entirely digital, and the implants did not undergo electropolishing, so they had a porous surface. In fact, the manufacturing of implants by DMLS, together with the treatment with organic acids, determine the formation of a porous surface, with concavities that continue in a structure with interconnections between the pores [29, 48–51]. This porosity, although usually lower than that generated by other AM procedures such as the electron beam melting (EBM) [52] has the potential to stimulate bone ingrowth [29, 48, 49], as well as soft tissue adhesion [50, 51], for better healing and long-term tissue stability.

Naturally, the present full digital technique has some problems. For the full digital design of these implants, experience and knowledge of CAD software is required. In particular, the thresholding is delicate for creating the 3D bone model on which the implant will be designed. In fact, if the thresholding is wrong, problems will inevitably arise in the fit and adaptation of the implant during the operation. It is therefore clear how, in the presence of CBCT with artefacts or substantial scattering in the area to be reconstructed, criticalities can emerge in the design of the implant, which in any case is not simple and requires adequate CAD knowledge. Some company services now offer help for the surgeon, at relatively low costs; these companies own the software and machines necessary for manufacturing the implants, and in this sense they represent the best solution for the clinician. In any case, surgery for placement of a subperiosteal implant is technically more complex than the classic positioning of endosseous implants *ad modum* Brånemark. The clinician’s surgical skills play a fundamental role in the insertion and in the management of any biological and technical complications.

This study has limits. Firstly, it is just a case series; a larger sample of patient would be advisable to draw more specific conclusions about the reliability of these implants. Secondly, this study has a short follow-up of 1 year, and therefore does not report on the possible mid- or long-term complications that may affect these custom-made subperiosteal implants. For example, the progressive atrophy of the bone in elderly patients could determine the mobilization in the implant, in the medium or long period. Extending the follow-up is therefore mandatory, as it could lead to more solid conclusions.

## Conclusions

In the past, subperiosteal implants represented a possible solution for prosthetic restoration of the atrophic posterior mandible. However, their use presented several technical and surgical issues (including the need for two surgical sessions, and often poor adaptation to the surgical site) that were in part responsible for the high percentage of complications in the short and long term.

Today, each patient must be examined in their complexity, evaluating the best solution considering every aspect, physiological, pathological, aesthetic and economic. Digital technologies such as DMLS allow revisiting the old concept of subperiosteal implants in a modern way, for treatment of the posterior atrophic mandible of elderly patients who do not want to undergo to bone regenerative procedures.

In the present study, ten elderly patients were treated with custom-made 3D-printed subperiosteal titanium implants. During surgery, the fit of the implants was satisfactory. Only two implants had insufficient fit, because of the presence of scattering in the CBCT; however, these were adapted to the surgical site during the intervention. The mean duration of the intervention was 44.3 min. At the one-year follow-up, no implants were lost, with a low incidence of complications that were minor in nature.

Although this case series has limits, such as the limited patient sample and the short follow-up, we can still draw positive conclusions. DMLS has proven to be an effective method to fabricate accurate subperiosteal implants with high success rates in elderly patients with severe bone atrophy, avoiding long and invasive regenerative therapies that are not possible in these patients. Furthermore, the complications were few, both in the postoperative time and at follow-up. We can therefore define the subperiosteal implants performed with DMLS as a possible alternative option for implant-prosthetic rehabilitation in elderly patients with bone atrophy, where inserting endosseous fixtures is not possible. To confirm these positive preliminary clinical outcomes, prospective clinical studies on a larger sample of patients and with longer follow-up are needed.

## Data Availability

All data generated during this study are available from the corresponding author, upon approval of all the authors of the manuscript.

## Abbreviations

.STL: Stereolithographic
3D: Three-dimensional
AM: Additive manufacturing
CAD: Computer-assisted-design
CAD/CAM: Computer-assisted-design/ computer-assisted manufacturing
CBCT: Cone beam computed tomography
CI: Confidence intervals
CT: Computerized tomography
DICOM: Digital imaging and communication in medicine
DMLS: Direct metal laser sintering
FOVs: Field-of-views
GBR: Guided bone regeneration
OPT: Orthopantomography
PMMA: Polymethyl-methacrylate
SD: Standard deviations
